# Degradation Mechanisms of Epoxy Coatings and Their Adhesion to Cementitious Substrates Under Intense Ultraviolet Radiation

**DOI:** 10.3390/polym18182216

**Published:** 2026-09-11

**Authors:** Binqiang Sun, Chao Xie, Wenzhe Ma, Chengkuo Liu

**Affiliations:** 1 Gansu Yi’an Construction Technology Group Co., Ltd., Lanzhou 730060, China; sbqgsya@163.com; 2 Civil Engineering Department, Lanzhou Jiaotong University, Lanzhou 730070, China

**Keywords:** UV aging, adhesion strength, characteristic functional groups, coating surface polarity, nano-adhesion force, coating toughness

## Abstract

To further reveal the degradation mechanism of epoxy coatings under intense ultraviolet radiation in high-altitude environments and clarify its influence on their interfacial adhesion performance, an epoxy coating-cement mortar system was investigated. Ultraviolet (UV) aging tests were conducted, together with attenuated total reflectance Fourier transform infrared spectroscopy (ATR-FTIR), surface free energy (SFE) measurements, atomic force microscopy (AFM)-based nano-adhesion force measurements, scanning electron microscopy and energy-dispersive X-ray spectroscopy (SEM/EDS), uniaxial tensile tests, and pull-off adhesion strength tests to investigate the evolution of the coating’s characteristic molecular structure, surface polarity, nano-adhesion, coating toughness, macroscopic adhesion performance, and interfacial failure modes at different aging stages. The results showed that the peaks associated with hydroxyl and carbonyl groups in the epoxy coating intensified with increasing UV aging duration. The surface free energy of the coating increased, and its polar component reached 3.9 times the initial value. After 28 d of UV aging, the nano-adhesion force of the coating decreased by 30.1%, its toughness decreased from 2.82 ± 0.052 to 1.21 ± 0.027 MJ·m^−3^, and the adhesion strength between the epoxy coating and the cementitious substrate decreased by 15.7%. In addition, as aging progressed, the failure path gradually shifted from fracture near the substrate surface toward regions near the coating–cementitious substrate interface and within the coating. Correlation analysis further showed that the decreases in coating toughness and nano-adhesion performance were closely associated with the deterioration of macroscopic adhesion strength. Therefore, greater attention should be paid to the optimization of these two properties in practical applications.

## 1. Introduction

Concrete has become one of the principal structural materials used in bridges, roads, tunnels, hydraulic facilities, and other infrastructure. As infrastructure construction gradually extends into cold, high-altitude regions and areas with high concentrations of corrosive media, concrete structures are exposed to increasingly complex service conditions. Long-term exposure to moisture and aggressive ions can adversely affect their durability [1,2]. To retard the penetration of external media into concrete, surface-applied protective materials are commonly used in engineering practice to form a protective barrier, thereby slowing structural deterioration, reducing subsequent maintenance costs, and extending service life [3,4]. This approach contributes to meeting the requirements of engineering construction for enhanced durability and low-carbon development.

Epoxy coatings are characterized by dense film formation, good adhesion, and high resistance to the penetration of aggressive media, enabling them to form a continuous protective barrier on cementitious substrates [5,6]. They have therefore been widely used to protect concrete structures exposed to the harsh environments of western China [7,8]. However, some engineering structures in western China are located in high-altitude plateau regions. Owing to the low air density and the reduced capacity of the atmosphere to scatter and absorb ultraviolet radiation, the UV radiation level at the ground surface is considerably higher than that in low-altitude plain regions; the annual average daily UV radiation dose can reach 0.595 MJ/m^2^/d [9,10]. Long-term exposure to UV radiation can induce photo-oxidative aging of epoxy coatings, resulting in changes in carbonyl, hydroxyl, amide, and other structures within the cured network [11,12,13,14]. This process may also be accompanied by molecular chain scission, the accumulation of oxidation products, and further cross-linking [15,16,17]. The resulting hardening and embrittlement of the coating weaken its toughness and deformation compatibility [18,19], thereby reducing the reliability of the bond between the coating and the cementitious substrate and leading to interfacial debonding [20,21,22]. Ultimately, these changes adversely affect the protective effectiveness of the coating and the service life of the structure [23,24].Extensive studies have been conducted worldwide on the UV aging of epoxy coatings. Liu et al. [25] investigated the UV aging behavior of polyamide-cured epoxy coatings using ATR-FTIR spectroscopy, slow positron annihilation spectroscopy, and electrochemical impedance spectroscopy. They found that molecular chain scission induced by photo-oxidation promoted the formation of microporous structures in the surface layer, further altering the moisture transport and barrier properties of the coating. Khotbehsara et al. [26] investigated the UV aging behavior of particle-filled epoxy coatings in terms of surface morphology, chemical structure, and thermodynamic properties. Their results showed that UV exposure caused surface yellowing, mass loss, and microcracking in the epoxy coating. Szociński et al. [27] divided the UV aging process of epoxy coatings into three stages—stability, degradation initiation, and failure—based on Raman spectroscopy, AFM morphological characterization, and electrochemical impedance spectroscopy. Wang et al. [28] investigated the UV aging of epoxy and polyurea protective coatings for concrete. Through analyses of surface morphology, pore structure, wettability, and chemical structure, they demonstrated that the decline in coating protection performance was closely related to deterioration of the coating itself and the loss of adhesion between the coating and concrete. Li et al. [29] investigated the chloride-ion barrier performance of different coatings under natural weathering and accelerated UV-wet/dry cyclic aging. They found that the resistance of the coatings to chloride-ion penetration decreased with aging and that their effective protection periods were closely related to the coating type and degree of aging. In summary, existing studies have primarily focused on changes in the surface color, gloss, morphology, and chemical functional groups of coatings. However, systematic analyses of the coating microstructure, degradation of mechanical properties, and the mechanisms through which these changes affect coating adhesion remain limited. Therefore, elucidating the aging behavior of epoxy coatings under intense UV radiation and the deterioration behavior and mechanisms of their adhesion performance with cementitious substrates has become an urgent issue in the protection of concrete structures exposed to intense UV radiation in high-altitude plateau environments.

Accordingly, an epoxy coating-cement mortar system was investigated to systematically examine the evolution of the coating structure, mechanical properties, and adhesion performance under intense UV radiation. ATR-FTIR spectroscopy, contact angle measurements, surface free energy measurements, and AFM tests were employed to analyze changes in the characteristic functional groups, surface polarity, and nano-adhesion force of the coating. Coating tensile tests and pull-off tests were conducted to evaluate the coating toughness and the adhesion strength between the coating and the cementitious substrate, respectively. SEM and EDS analyses were further conducted to characterize the microstructural features and elemental distributions of the fracture surfaces after bond failure between the coating and the cementitious substrate. Through this multiscale approach, the deterioration behavior of the coating under intense UV radiation and the mechanism by which such deterioration affects the adhesion performance between the coating and the cementitious substrate were systematically elucidated, providing a theoretical basis and technical support for evaluating the durability of protective coatings for concrete in high-altitude regions exposed to intense UV radiation.

## 2. Experimental Program

### 2.1. Raw Materials and Specimen Preparation

For the pull-off tests, P.O. 42.5 ordinary Portland cement supplied by Xinjiang Tianshan Cement Co., Ltd. (Urumqi, Xinjiang, China) was used. Its flexural and compressive strengths were 7.8 and 47.3 MPa, respectively. The mass ratio of cement, sand, and water used to prepare the mortar was 1:2.5:0.4. Mortar specimens measuring 70 mm × 70 mm × 20 mm were prepared. The specimens were subjected to standard curing (at a relative humidity of 95% and a temperature of 20 ± 2 °C) for 28 d and then stored until further use. After curing, the laitance layer on the mortar surface was removed using a grinding machine. Surface dust was subsequently removed, and the specimens were dried at room temperature to minimize the influence of the cement mortar surface condition on the coating adhesion strength.

**Figure 1:**
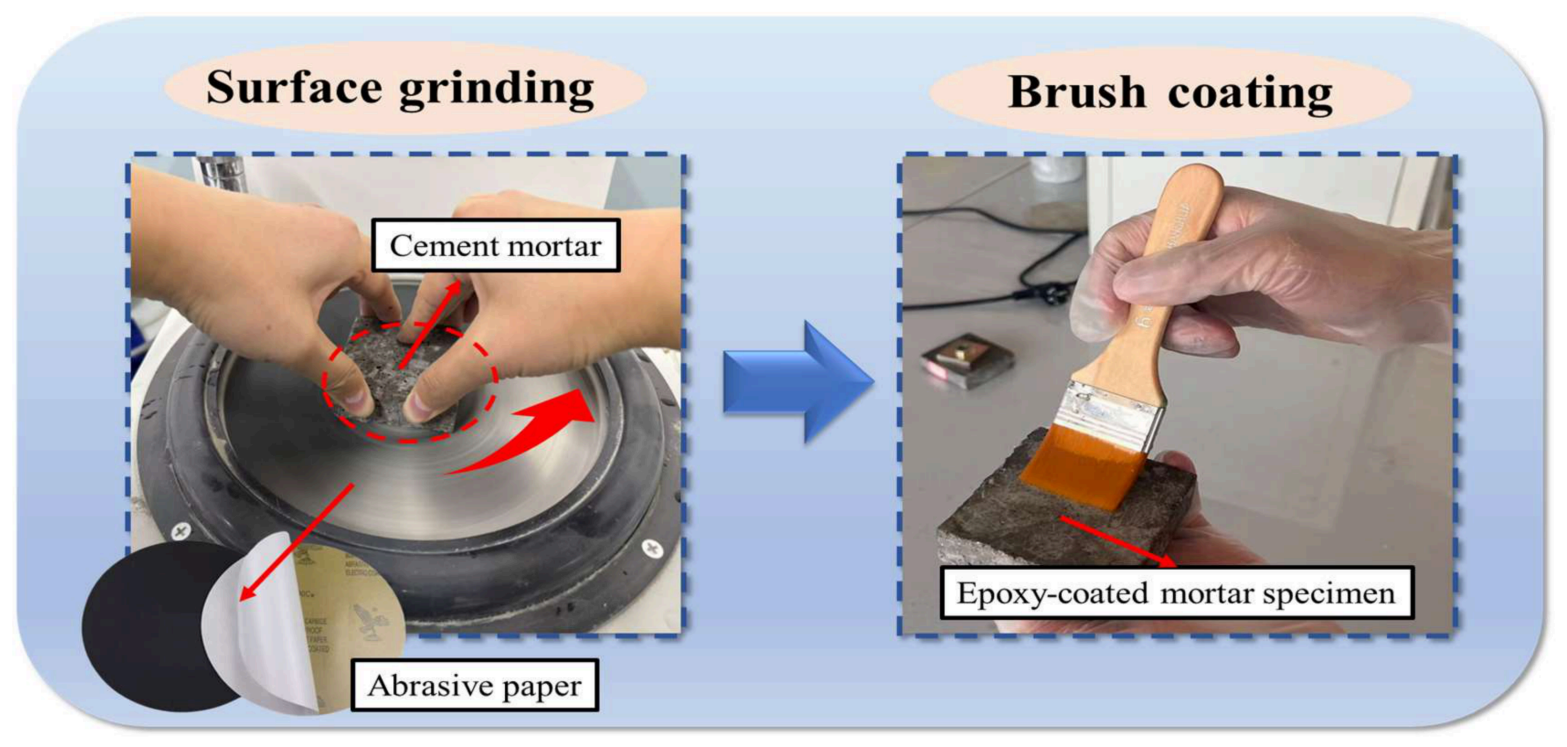
Preparation of epoxy coating-cement mortar specimens.

**Figure 2:**
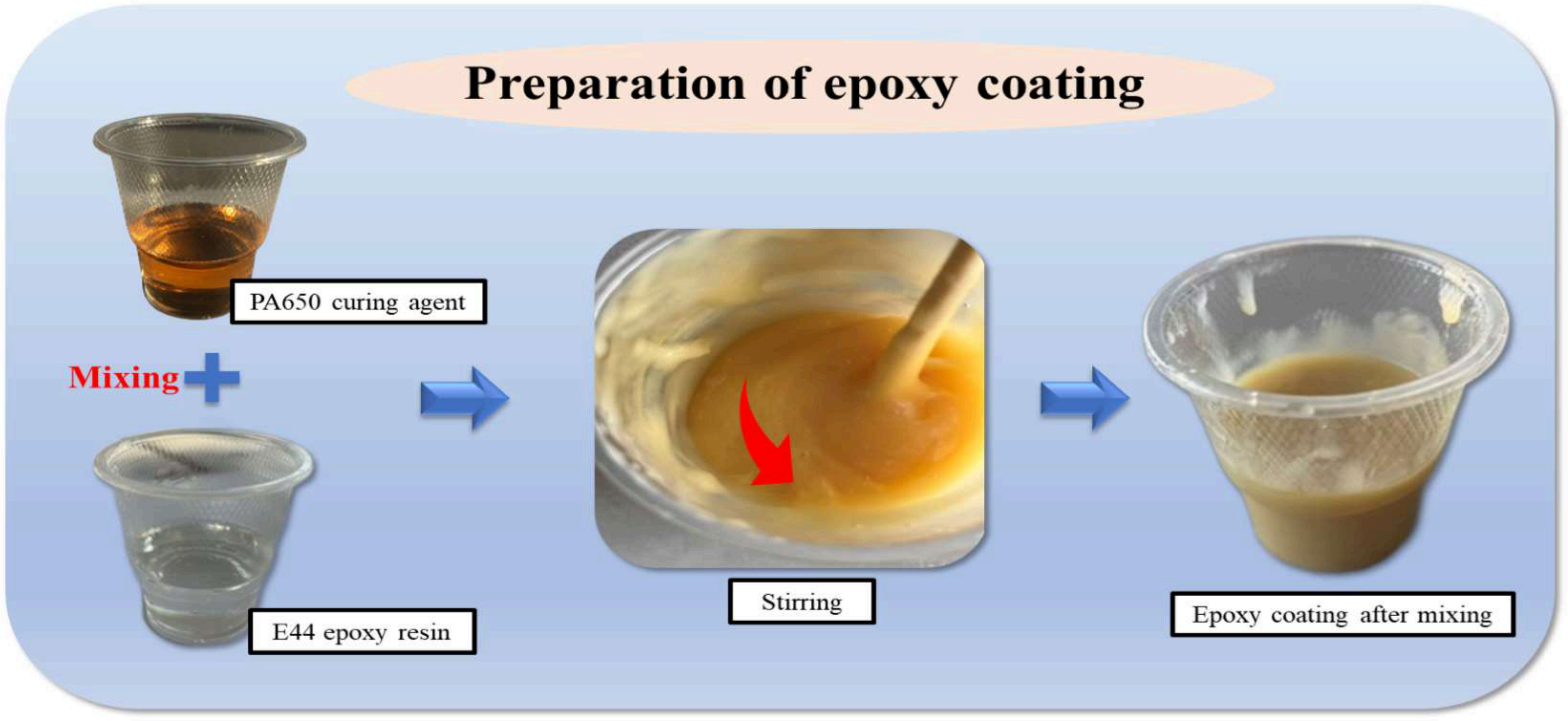

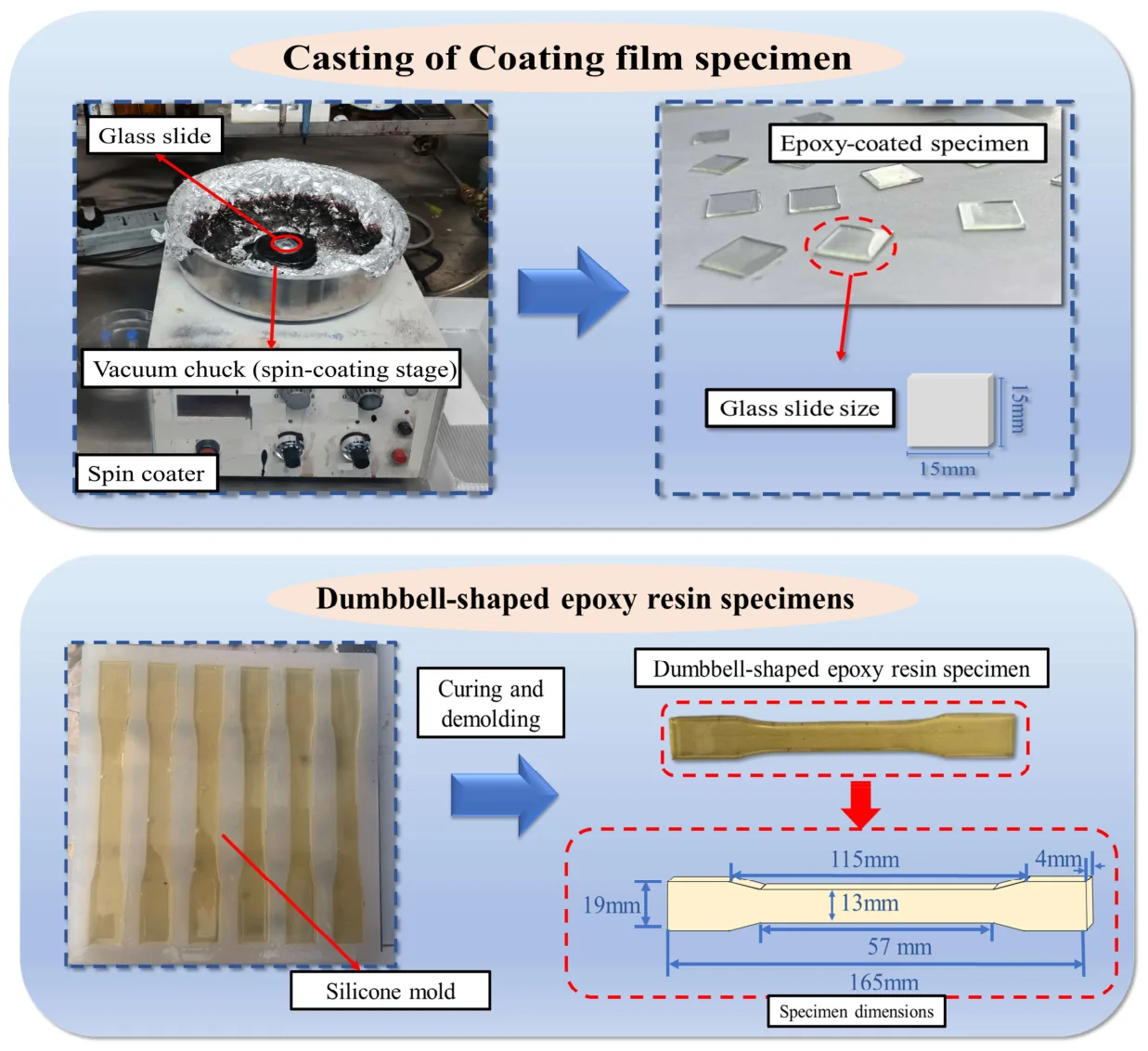
Preparation of epoxy coating film specimens and dumbbell-shaped tensile specimens.

The coating material used in this study was E44 epoxy resin supplied by Ailik New Materials Co., Ltd. (Shenzhen, Guangdong, China) (with a matching polyamide 650 curing agent), and its main performance parameters are listed in Table 1. The E44 epoxy resin and polyamide 650 curing agent were thoroughly mixed at a mass ratio of 1:1. The mixture was then thoroughly stirred for 0.5 h. The prepared epoxy coating material was subsequently applied uniformly to the surfaces of the cement mortar specimens. After the coating had fully cured, the specimens were used for subsequent tests of the adhesion strength between the coating and the cementitious substrate and for surface free energy measurements. For the adhesion strength test, three parallel specimens were prepared at each aging duration (*n* = 3 specimens), whereas for the surface free energy test, one coating–cement mortar specimen was selected at each aging duration for one measurement (*n* = 1 measurement). The specimen preparation procedure is shown in Figure 1. For testing the properties of the coating itself, epoxy coating specimens measuring 15 mm × 15 mm were prepared using a KW-4A spin coater for ATR-FTIR spectroscopy, contact angle measurements, and AFM tests. One independent specimen was prepared at each aging duration (*n* = 1 specimen). Meanwhile, dumbbell-shaped epoxy tensile specimens measuring 165 mm × 19 mm × 4 mm were prepared with reference to ASTM D638-22 [30] to evaluate changes in the tensile properties and toughness of the coating at different UV aging durations. Three parallel tensile specimens were prepared for each aging duration (*n* = 3 specimens). The specimen preparation procedure is shown in Figure 2. All specimens were cured at 40 °C for 4 h and then at 60 °C for 20 h, followed by further curing at room temperature (25 °C) for 7 d [31] before UV aging treatment, to minimize the influence of differences in the degree of curing on the test results.

### 2.2. UV Aging Test Method

**Figure 3:**
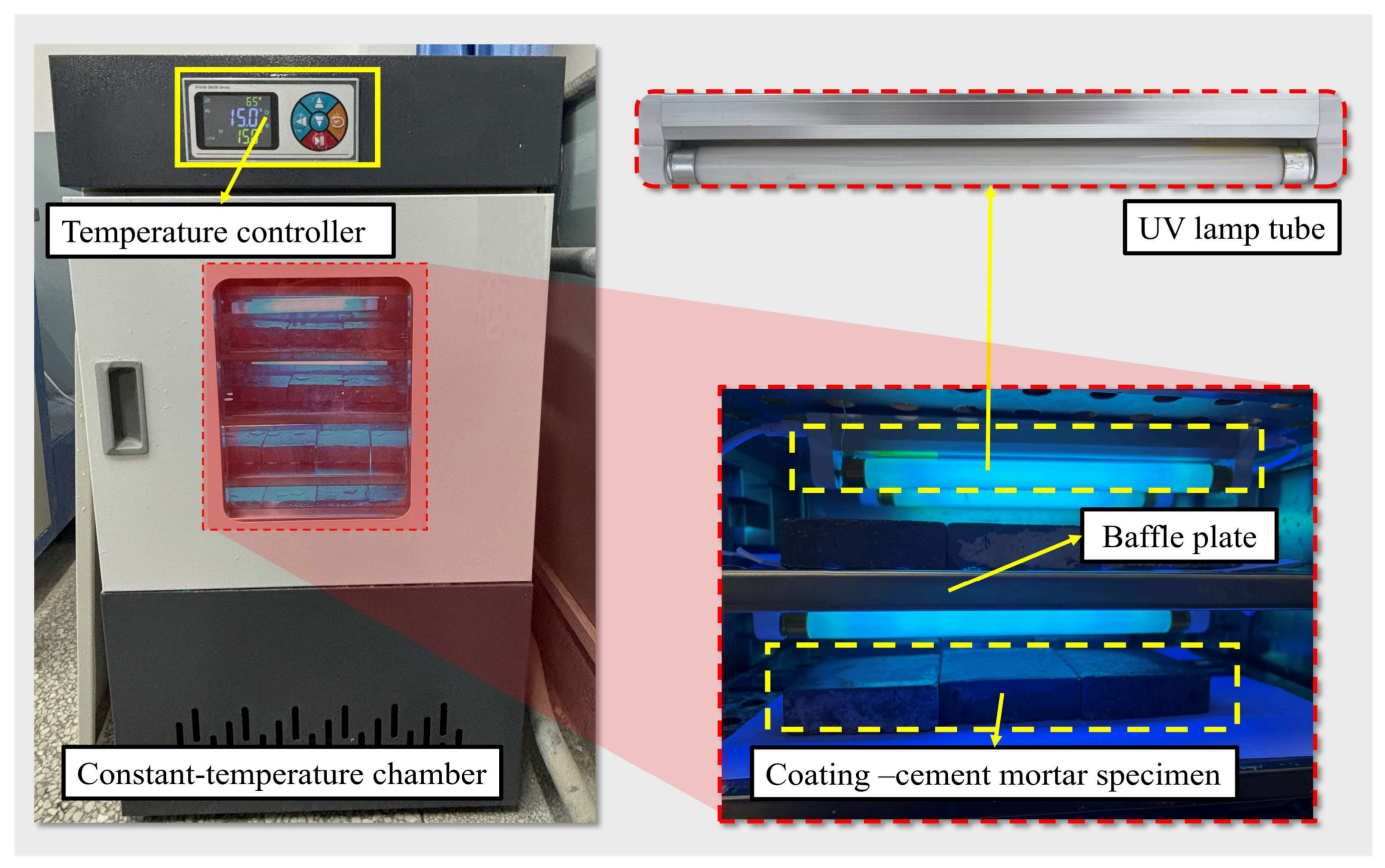
UV aging test apparatus for epoxy coatings.

The UV aging test was conducted in a temperature-controlled chamber, as shown in Figure 3. With reference to ASTM D4587-23 [32], fluorescent UV lamps were installed in the chamber, with a peak wavelength of 340 nm. The 340 nm UV light source was primarily used to simulate the photoaging effect of short-wavelength UVA radiation in sunlight on organic coatings [33]. Referring to the annual average UV irradiance of 20 W/m^2^ in high-altitude regions, this value was adopted as the irradiation intensity in the present study [34,35]. With reference to the environmental characteristics of the plateau regions of western China, including low temperatures, long sunshine duration, and intense UV radiation, the aging temperature was set to 15 °C [36], and the daily UV aging duration was 10 h [37]. The specimens were removed after aging for 0 d, 7 d, 14 d, and 28 d and subsequently subjected to ATR-FTIR, SFE, AFM, tensile, and pull-off tests. The present study aimed to investigate the effects of intense UV aging on the intrinsic properties of the epoxy coating and its adhesion performance with the cementitious substrate. To more clearly elucidate the evolution of the coating microstructure, mechanical properties, and adhesion characteristics under intense UV radiation, as well as the influence of changes in the intrinsic coating properties on its adhesion to the cementitious substrate, variations in environmental humidity were not considered in the present experimental design. Therefore, the coupled effects of intense UV radiation and humidity were beyond the scope of this study, which represents a limitation of the present work. The influence of environmental humidity will be incorporated in future studies to further investigate the coupled deterioration behavior.

### 2.3. Experimental Methods

#### 2.3.1. Attenuated Total Reflectance Fourier Transform Infrared Spectroscopy (ATR-FTIR)

A Thermo Nicolet iS5 Fourier (Thermo Electron Scientific Instruments LLC, Madison, WI, USA) transform infrared spectrometer was used to characterize the molecular structure of the epoxy coatings at different UV aging durations. The measurements were performed in attenuated total reflectance mode over a wavenumber range of 4000–500 cm^−1^ at a resolution of 4 cm^−1^. Each sample was scanned three times, and the resulting spectra were averaged (3 scanned regions per specimen). Baseline correction and smoothing of the obtained spectra were performed using OMNIC 8.2 software. Changes in the bands associated with amine, carbonyl, hydroxyl, and other groups were analyzed to evaluate the effects of UV aging on the functional groups and molecular structure of the epoxy coating.

#### 2.3.2. Atomic Force Microscopy (AFM)

A Multimode 8 atomic force microscope (Bruker, Santa Barbara, CA, USA) operated in PeakForce Tapping quantitative nanomechanical mapping mode (PFT-QNM) was used to characterize the surface morphology and nano-adhesion force of the coating. A Bruker RTESPA-150 (Bruker AFM Probes, Camarillo, CA, USA) probe with a nominal tip radius of 8 nm and a deflection sensitivity of 85 nm/V was used. The test parameters were set as follows: scanning frequency, 1.0 Hz; scanning area, 5 μm × 5 μm; resolution, 256 × 256 pixels; cantilever spring constant, 5 N/m; and test temperature, 25 °C. The data were analyzed using the DMT contact mechanics model, and Nano-adhesion was determined from the pull-off force in the retraction segment of the force–distance curve. Three different areas were scanned for each specimen at each aging duration (*n* = 3 scanned regions per specimen) to minimize the influence of surface heterogeneity on the test results. Nano Scope Analysis software (version 1.5, Bruker, Santa Barbara, CA, USA) was used to statistically analyze the valid Nano-adhesion data within each scanned region and to calculate the mean Nano-adhesion value for each region. These results were used to evaluate the effect of UV aging on the local adhesion properties of the epoxy coating.

#### 2.3.3. Surface Free Energy (SFE)

A DataPhysics OCA 25 optical contact angle goniometer (DataPhysics Instruments GmbH, Filderstadt, Germany) was used to measure the contact angles of three probe liquids—deionized water, formamide, and diiodomethane—on the coating and substrate surfaces. The measurements were conducted at a room temperature of 25 °C and a relative humidity of 60%. At each aging duration, one specimen (*n* = 1 specimen) was selected for the surface free energy test, and one contact angle measurement was performed for each probe liquid on the specimen surface (1 measurement per liquid). The surface free energy was calculated using Equation (1) based on the Owens-Wendt-Rabel-Kaelble (OWRK) method [38,39,40]. For the overdetermined system obtained from the three probe liquids, the OWRK equation was linearized by defining $\sqrt{\gamma_{L}^{p}/\gamma_{L}^{d}}$ as the independent variable and $\gamma_{L}(1+\cos\theta )/2\sqrt{\gamma_{L}^{d}}$ as the dependent variable. The results obtained using deionized water, formamide, and diiodomethane were fitted by least-squares linear regression. The intercept and slope of the fitted line correspond to $\sqrt{\gamma_{S}^{d}}$ and $\sqrt{\gamma_{S}^{p}}$, respectively. Thus, the Lifshitz–van der Waals component $\gamma_{S}^{d}$ and polar component $\gamma_{S}^{p}$ of the coating surface free energy were determined, and the total surface free energy $\gamma_{S}^{\mathrm{Total}}$ was calculated as the sum of these two components [41].

(1) $$\gamma_{L}\left(\cos\theta_{L}+1\right)=2\left(\sqrt{\gamma_{L}^{d}\gamma_{S}^{d}}+\sqrt{\gamma_{L}^{p}\gamma_{S}^{p}}\right)$$

where $\gamma_{L}$ is the total surface tension of the probe liquid; $\theta_{L}$ is the contact angle of the probe liquid on the solid surface; $\gamma_{L}^{d}$ and $\gamma_{L}^{p}$ are the Lifshitz–van der Waals and polar components of the surface tension of the probe liquid, respectively; and $\gamma_{S}^{d}$ and $\gamma_{S}^{p}$ are the Lifshitz–van der Waals and polar components of the surface free energy of the solid, respectively.

#### 2.3.4. Tensile Test

**Figure 4:**
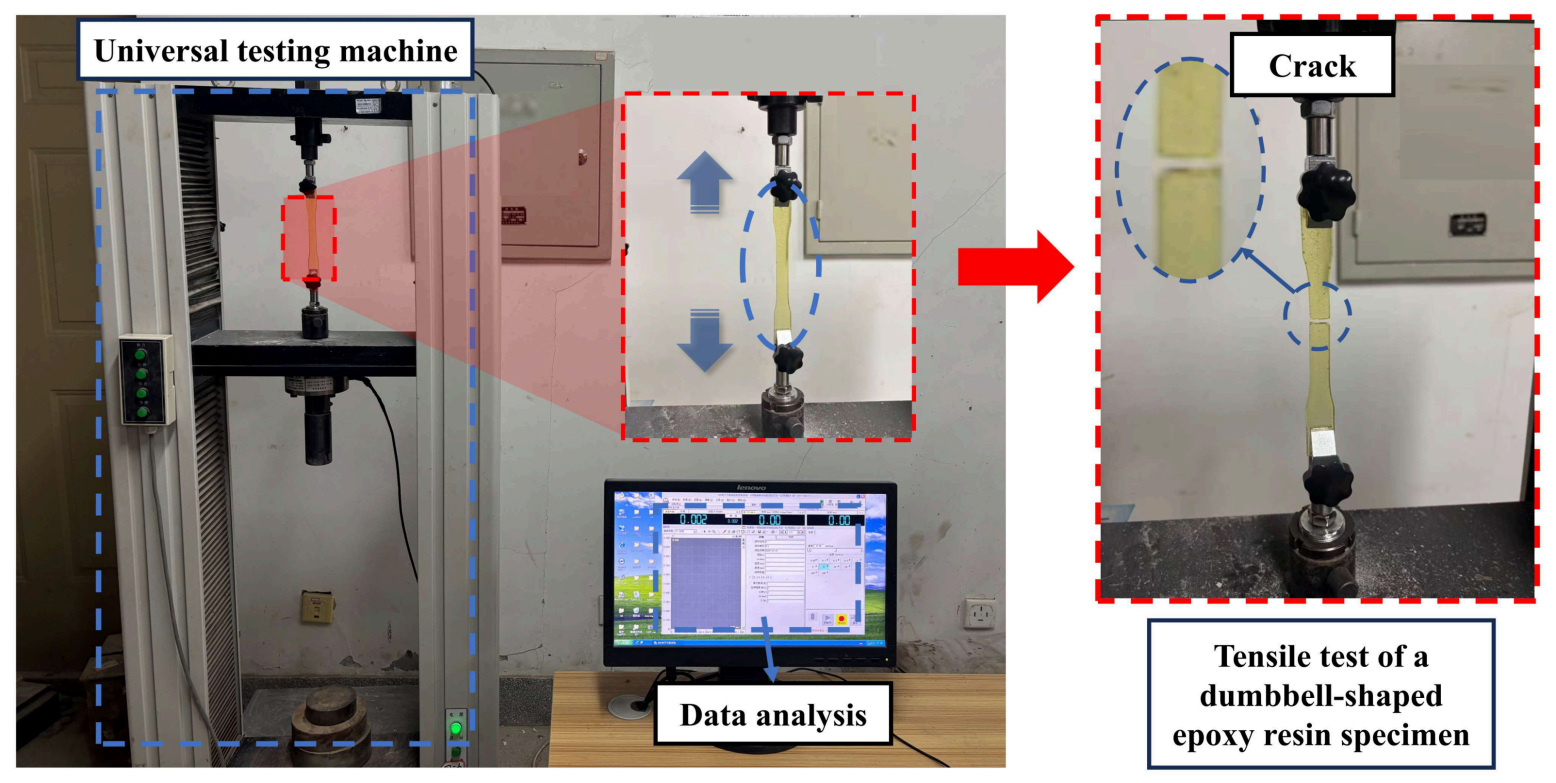
Tensile test setup for epoxy coatings.

Tensile tests were conducted using a universal testing machine in accordance with GB/T 2567-2021 [42], as shown in Figure 4. The measurement accuracies were ±1% for load and ±0.5% for displacement, and the loading rate was 2 mm/min. The maximum tensile strength, *σ*_t_, of the coating at different aging durations was measured, and the coating toughness was calculated from the corresponding stress–strain curves using Equation (2).

(2) $$Τ=\int_{0}^{\varepsilon_{b}}\sigma_{t}(\varepsilon )d\varepsilon$$

where τ is the toughness; $\sigma_{t}(\varepsilon )\;$ is the stress as a function of strain; ε is the strain; and $\varepsilon_{b}\;$ is the strain at break.

#### 2.3.5. Pull-Off Test

**Figure 5:**
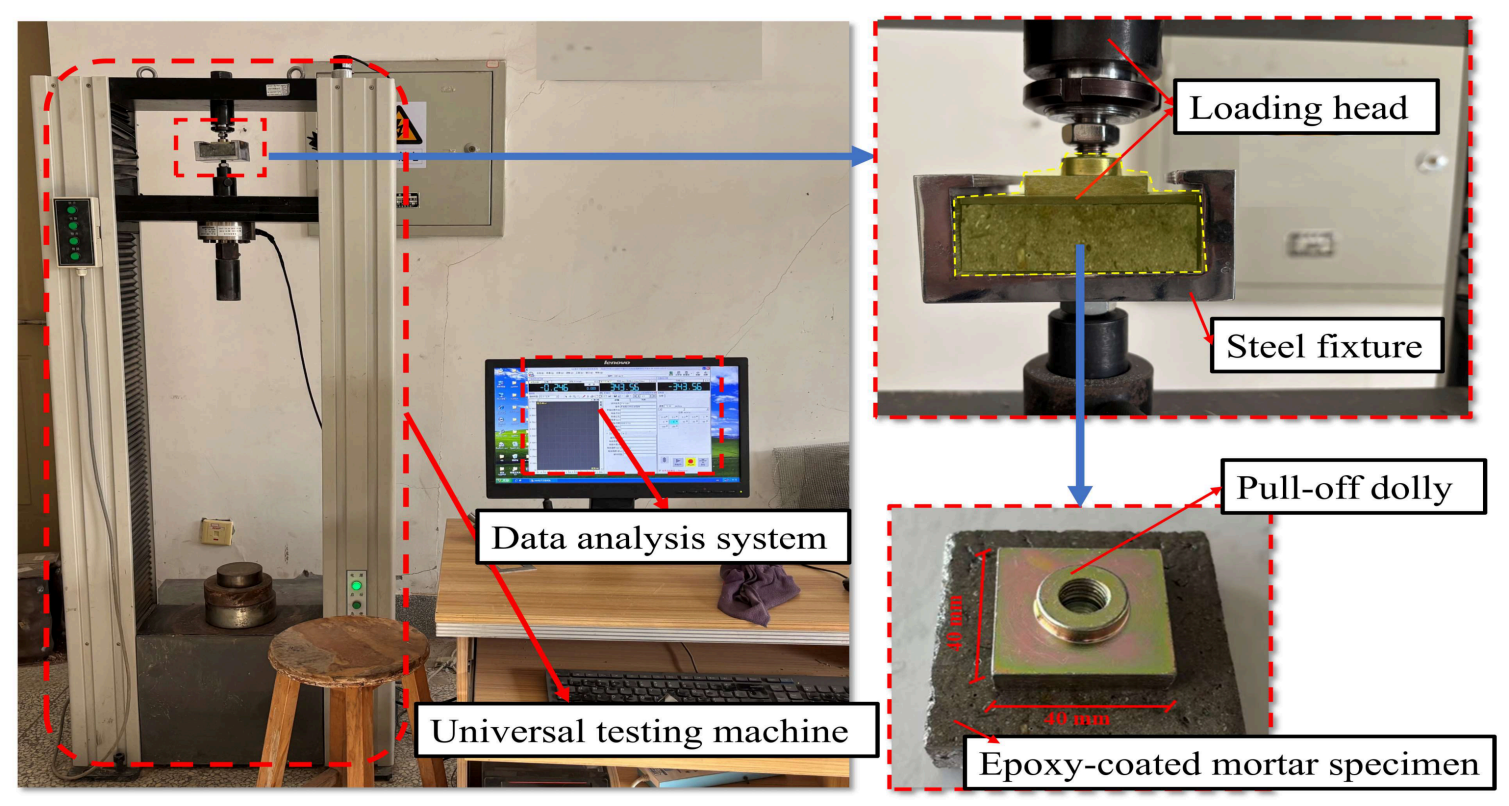
Pull-off test setup for the epoxy coating-cementitious substrate system.

The adhesion strength between the epoxy coating and the cement mortar substrate was measured using the vertical pull-off method. The test was conducted in accordance with Method A of GB/T 16777-2008 [43], as shown in Figure 5. Before testing, a 40 mm × 40 mm pull-off dolly with an effective bonding area of 1600 mm^2^ was bonded to the coating surface using a two-component epoxy adhesive, with components A and B mixed at a mass ratio of 1:1. After the adhesive had fully cured, the coating was cut through to the substrate along the perimeter of the pull-off dolly. A tensile load was applied perpendicular to the coating surface using a universal testing machine at a loading rate of 5 mm/min until failure of the coating–cement mortar system occurred. The adhesion strength was calculated using Equation (3):

(3) $$\sigma_{A}=\frac{F_{p}}{A}$$

where $\sigma_{A}$ is the adhesion strength between the coating and the cement mortar substrate (Mpa); *F*_p_ is the maximum pull-off load at specimen failure (N); and *A* is the effective bonded area between the pull-off dolly and the coating (mm^2^).

#### 2.3.6. SEM Morphology and EDS Elemental Analysis

JSM-7610Plus (JEOL Ltd., Tokyo, Japan) scanning electron microscope (SEM) was used to characterize the bond failure characteristics of the coating–cementitious substrate system and the evolution of the interface at different UV aging durations. The observations were conducted in secondary electron detector (SED) mode under high-vacuum conditions, with an accelerating voltage of 10.0 kV and a working distance of approximately 12.2–14.1 mm. Typical interfacial morphologies were observed at a magnification of 200×.

## 3. Results and Discussion

### 3.1. Characteristic Functional Groups of the Coating

Interactions between polar sites on the surfaces of cement hydration products and oxygen- and nitrogen-containing groups in the epoxy resin constitute one of the principal contributions to bonding between the cementitious substrate and the epoxy coating [44]. Polar functional groups in the epoxy resin, including carbonyl, hydroxyl, and amine groups, can participate in interfacial bonding through hydrogen bonding, electrostatic interactions, and van der Waals interactions [45]. Therefore, changes in these functional groups and their molecular chain structures directly affect the interfacial adhesion performance between the epoxy coating and the cementitious substrate.

**Figure 6:**
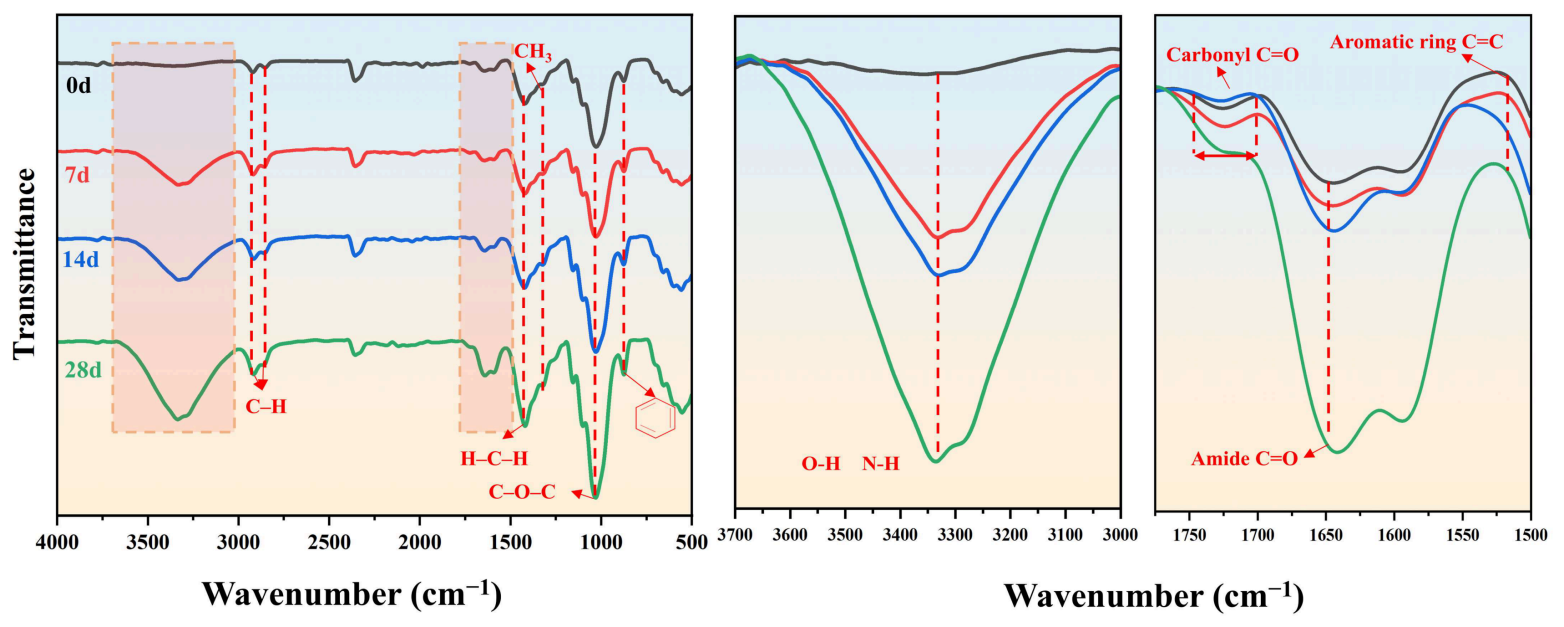
ATR-FTIR spectra of epoxy coatings at different UV aging durations.

The ATR-FTIR spectra of the epoxy resin coatings at different UV-aging durations are shown in Figure 6, in which several characteristic absorption bands can be identified. The bands at 2920 and 2850 cm^−1^ are assigned to the stretching vibrations of C–H bonds, while the band at 1462 cm^−1^ corresponds to the bending vibration of the H–C–H angle; these vibrations originate from CH_2_ groups in the cured epoxy network. In addition, several characteristic bands of bisphenol A-type epoxy resin are observed. Specifically, the band at 1510 cm^−1^ is attributed to the skeletal vibration of the aromatic ring (C=C), the band at 1362 cm^−1^ corresponds to the bending vibration of CH_3_, the band at 1038 cm^−1^ is assigned to the stretching vibration of the ether C–O–C bond, and the band at 830 cm^−1^ is associated with the benzene ring [46,47]. It should be noted that the sharp signal at approximately 2350 cm^−1^ mainly originates from residual atmospheric CO_2_ absorption during FTIR measurements [48].

To further elucidate the UV-aging process of the cured epoxy system, the variations in peak intensity and peak shape within the 3000–3600 cm^−1^ and 1500–1800 cm^−1^ regions were analyzed in detail [49]. As shown in Figure 6, the absorption intensities in both regions increase with increasing UV-aging time. The broad band centered at approximately 3350 cm^−1^ within the 3000–3600 cm^−1^ region is associated with O–H stretching vibrations of hydroxyl groups and N–H stretching vibrations of amine or amide groups in the cured epoxy network. The gradual enhancement of this band with prolonged UV exposure indicates an increase in oxygen-containing structures, including carboxylic acid groups [50]. The characteristic band near 1650 cm^−1^ is mainly attributed to the C=O stretching vibration of amide groups in the polyamide-cured system. With increasing UV-aging time, this band becomes broader and more intense, which is associated with the formation of oxidation products such as tertiary amides. Meanwhile, the carbonyl (C=O) absorption in the 1700–1750 cm^−1^ region, centered near 1725 cm^−1^, also becomes progressively stronger, indicating the gradual formation of oxygen-containing structures such as ketones and aldehydes during UV aging [11]. These spectral changes indicate that the degree of oxidation of the coating progressively increases with prolonged UV exposure.

During UV photo-oxidation, polar oxygen-containing functional groups such as hydroxyl and carbonyl groups are generated within the cured epoxy network [11,15]. This is attributed to the further decomposition of hydroperoxide intermediates formed during the photo-oxidation process [51], accompanied by the scission of molecular bonds associated with the bisphenol-A aromatic ether structure in the epoxy resin [47,52], thereby leading to the deterioration of the intrinsic mechanical properties of the coating [16,17] and a decrease in tensile strength. These changes are manifested by the gradual hardening and embrittlement of the coating, resulting in reduced toughness and deformation capacity [18,19]. These changes hinder the ability of the coating to deform compatibly with the cementitious substrate and dissipate interfacial stresses, making stress concentration and crack propagation more likely at interfacial defects and ultimately leading to deterioration of the adhesion performance between the epoxy coating and the cementitious substrate.

### 3.2. Surface Free Energy of the Coating

#### 3.2.1. Contact Angle of the Coating

**Figure 7:**
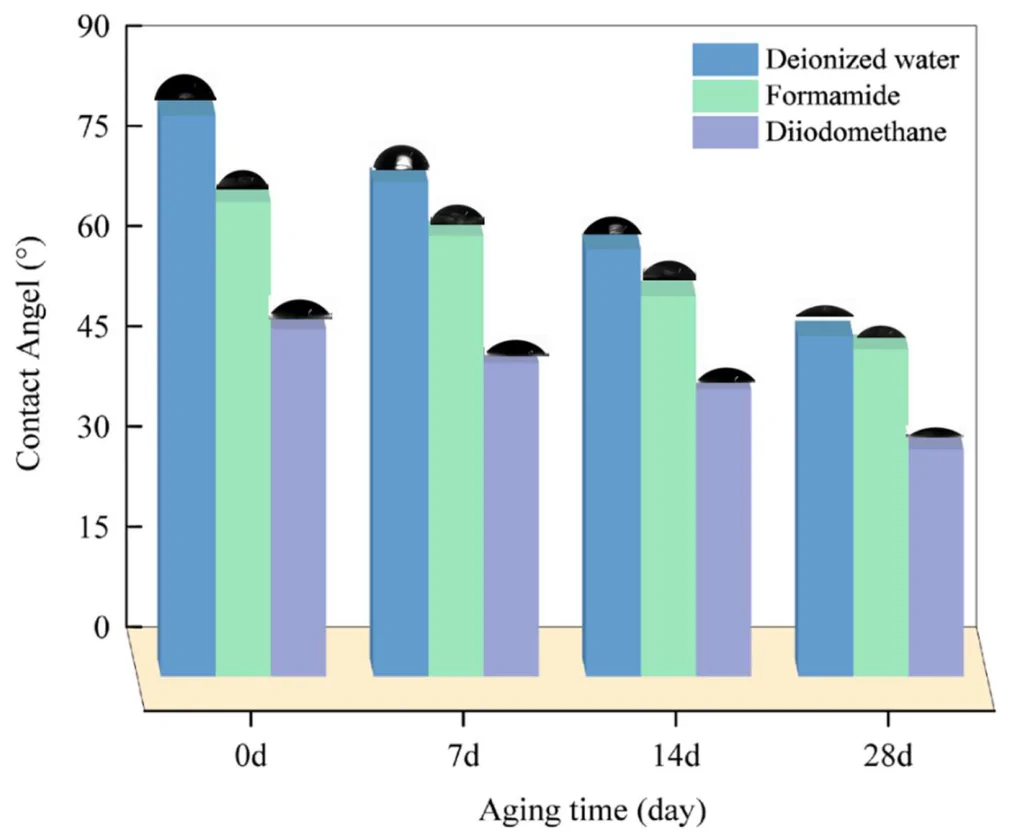
Contact angles of three probe liquids on epoxy coating surfaces at different UV aging durations.

With increasing UV aging duration, the contact angles of deionized water, formamide, and diiodomethane on the epoxy coating surface showed a continuous decreasing trend. The specific data are listed in Table 2, and the decreasing trends are shown in Figure 7, indicating that UV aging significantly altered the surface wettability of the coating. Specifically, the contact angle of deionized water decreased from 82° at 0 d to 51° at 28 d, that of formamide decreased from 71° to 49°, and that of diiodomethane decreased from 52° to 34°. After aging, the spreading ability of polar liquids on the coating surface increased, and the epoxy coating surface became progressively more hydrophilic. This change was mainly associated with the formation of polar oxygen-containing structures, such as hydroxyl and carbonyl groups, during UV-induced photo-oxidation [47]. Meanwhile, surface roughening and microdefects induced by UV aging may also promote the spreading of the probe liquids, thereby contributing to the reduction in contact angle [53].

#### 3.2.2. Surface Polarity of the Coating

**Figure 8:**
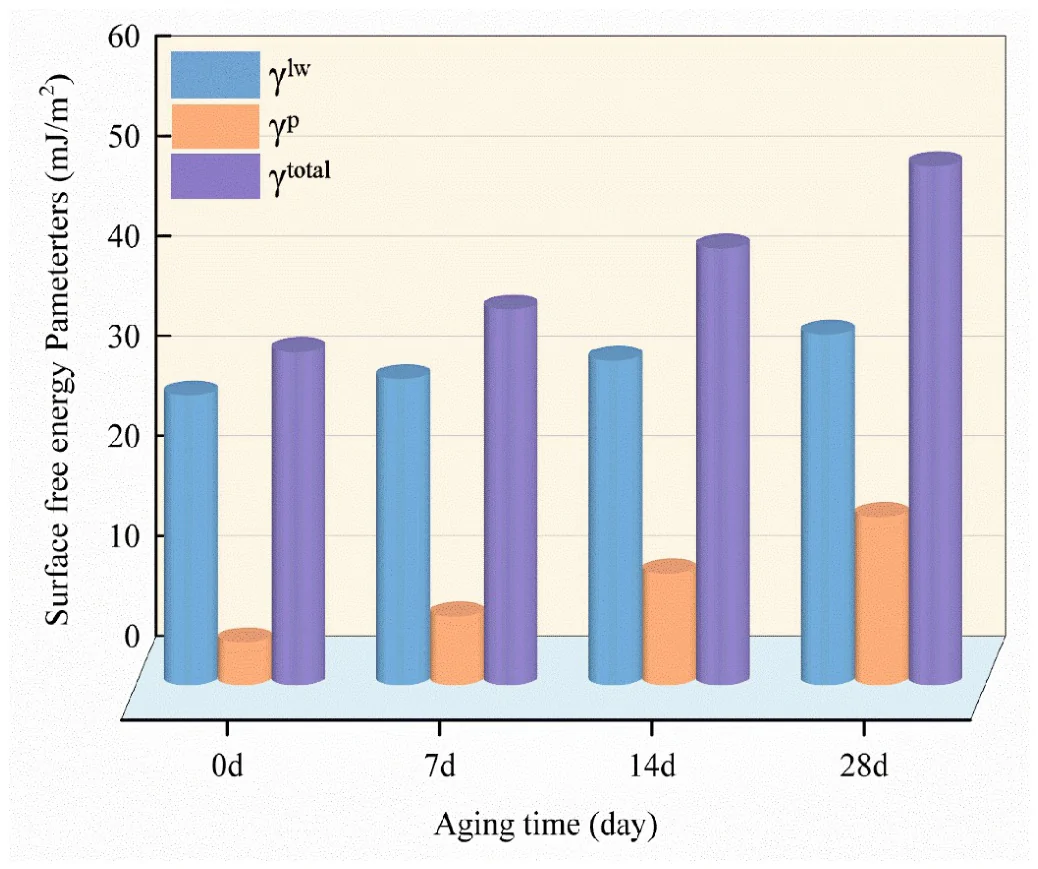
Surface free energy and its components of epoxy coatings at different UV aging durations.

The contact angle results for the three probe liquids, together with the corresponding liquid parameters listed in Table 3, were substituted into the OWRK model to calculate the surface free energy and its polar and dispersive components of the coating at different aging durations. The calculated results are listed in Table 4, and their variation trends are shown in Figure 8. With increasing UV aging duration, the dispersive component changed only slightly, whereas the polar component increased to 3.9 times its initial value. These results indicate that the surface polarity of the coating gradually increased, which is consistent with the aforementioned results for the characteristic functional groups. Combined with the preceding experimental results, the formation of polar structures, such as hydroxyl and carbonyl groups, enhanced the interaction between the coating surface and polar liquids. This finding further demonstrates that UV aging altered the characteristic molecular structure of the epoxy coating.

With increasing UV aging duration, polar oxygen-containing functional groups, such as hydroxyl and carbonyl groups, were gradually formed, increasing the surface polarity and hydrophilicity of the epoxy coating. However, this increased hydrophilicity facilitates moisture retention in defective regions of the coating, such as micropores and microcracks, and may allow aging-derived surface impurities to be carried along these defects into the coating interior and toward the coating-cementitious substrate interface [55]. On the one hand, water adsorbed at the interface can form a molecular water layer between the epoxy coating and the cementitious substrate, occupying the effective interaction sites of polar groups and weakening the original microscopic adhesive interactions [56]. On the other hand, moisture absorption can reduce the fracture toughness of the coating–cementitious substrate interface, making interfacial cracks more susceptible to propagation and debonding [57]. Therefore, under actual service conditions, the sensitivity of the coating–substrate interface to moisture may be further increased, thereby reducing the long-term service life of the coating and weakening its protective performance.

### 3.3. Nano-Adhesion of the Coating

**Figure 9:**
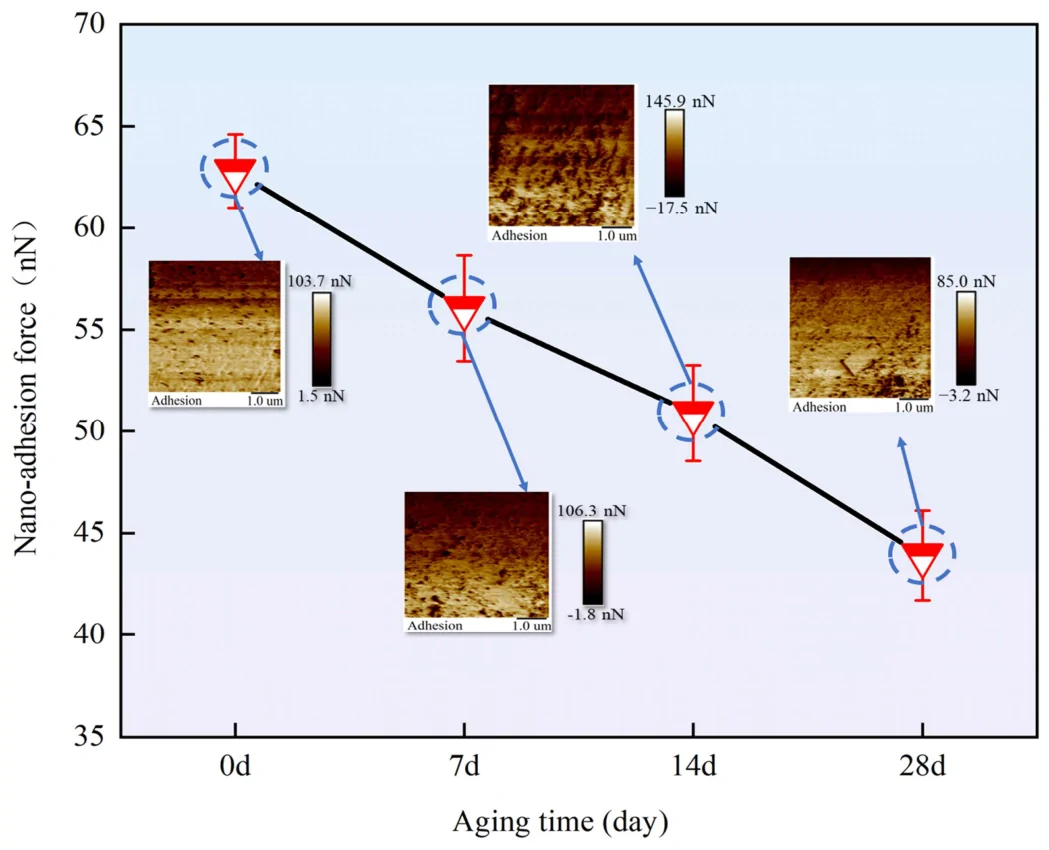
Nano-adhesion distributions and mean nano-adhesion of epoxy coatings at different UV aging durations.

The nano-adhesion of a coating reflects the combined effects of various microscopic adhesive interactions between the coating and the cementitious substrate, including chemical bonding, hydrogen bonding, and van der Waals interactions [58,59], and constitutes the basis of interfacial bonding between the coating and the substrate. The nano-adhesion distribution reflects the spatial distribution characteristics of the coating’s adhesion performance. The nano-adhesion results of the epoxy coating at different aging durations are presented in Figure 9, showing the evolution of the coating adhesion state and the high- and low-adhesion regions during UV aging. The Nano-adhesion maps shown in Figure 9 represent typical scanned regions, whereas the quantitative results are expressed as the mean ± standard deviation obtained from three scanned regions per specimen (3 scanned regions per specimen). As shown in the figure, the nano-adhesion distributions of the epoxy coating differed markedly among the different UV aging durations. In the unaged sample, the high-adhesion regions exhibited a relatively continuous distribution and good overall uniformity. With increasing aging duration, the high-adhesion regions gradually became more discrete, whereas the low-adhesion regions and locally heterogeneous regions increased. After 28 d of aging, the low-adhesion regions expanded further, indicating that UV aging progressively degraded the nano-adhesion performance of the epoxy coating and increased the spatial variability of its adhesion.

As shown in Figure 9 and Table 5, the mean nano-adhesion of the epoxy coating continuously decreased with increasing UV aging duration. The mean Nano-adhesion was 62.8 ± 1.80 nN for the unaged sample and decreased to 56.1 ± 2.60 nN, 50.9 ± 2.35 nN, and 43.9 ± 2.20 nN after 7, 14, and 28 d of aging, respectively. Compared with the unaged sample, the mean nano-adhesion decreased by 10.7%, 18.9%, and 30.1%, respectively, indicating that UV aging significantly weakened the nano-adhesion performance of the coating. Combined with the aforementioned ATR-FTIR and SFE results, the changes in the characteristic molecular structure and the accumulation of surface defects caused by aging jointly weakened the effective interactions between the probe and the coating, ultimately resulting in a continuous decrease in nano-adhesion. Furthermore, according to the weak boundary layer theory [60,61], when a coating is subjected to external degradation, debonding preferentially initiates in low-adhesion regions, subsequently triggering a cascading process that accelerates the overall debonding and delamination of the coating. This process reduces the interfacial adhesion performance between the coating and the cementitious substrate and shortens the service life of the coating [62,63]. Therefore, the deterioration of nano-adhesion during UV aging adversely affects the macroscopic interfacial adhesion performance of the coating.

### 3.4. Coating Toughness

**Figure 10:**
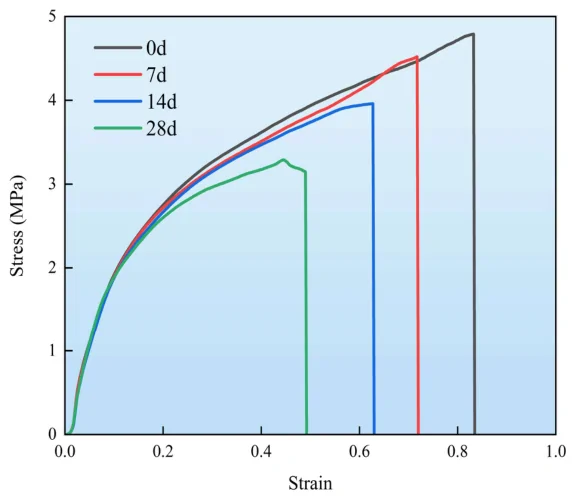
Tensile stress–strain curves of epoxy coatings at different UV aging durations.

The preceding results indicate that UV aging altered the characteristic molecular structure of the cured epoxy network, which was accompanied by changes in the mechanical properties of the coating. As shown in Figure 10, both the peak stress and strain at break of the epoxy coating continuously decreased with increasing UV aging duration. The detailed test results are listed in Table 6. The unaged coating exhibited a tensile strength of 4.79 ± 0.065 Mpa and a strain at break of approximately 0.83 ± 0.010. After 28 d of aging, the peak stress and strain at break decreased to 3.29 ± 0.045 Mpa and 0.445 ± 0.008, respectively. Compared with the unaged sample, the peak stress and strain at break of the sample aged for 28 d decreased by 31.3% and 46.4%, respectively, indicating that UV aging not only reduced the tensile load-carrying capacity of the epoxy coating but also severely weakened its deformation capacity.

**Figure 11:**
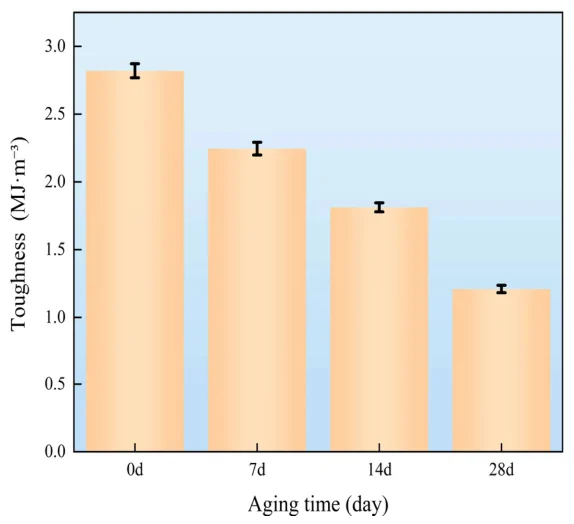
Toughness of epoxy coatings at different UV aging durations.

The coating toughness was calculated by integrating the stress–strain curve and reflects the ability of the coating to absorb and dissipate energy before fracture. As shown in Figure 11 and Table 6, the toughness of the epoxy coating gradually decreased from approximately 2.82 ± 0.052 MJ·m^−3^ in the unaged state to approximately 1.21 ± 0.027 MJ·m^−3^ after 28 d of aging, representing a reduction of 57.1%. This result indicates that UV aging significantly weakened the deformation load-carrying capacity and energy dissipation capacity of the coating before fracture. Combined with the aforementioned ATR-FTIR results, UV aging altered the characteristic molecular structure of the cured epoxy network. These structural changes were accompanied by reductions in the deformation and energy dissipation capacities of the coating, as evidenced by the decreases in strain at break and toughness, indicating progressive embrittlement of the coating [18,19]. Consequently, the ability of the coating to deform compatibly with the cementitious substrate and dissipate interfacial stresses was reduced, making stress concentration more likely at interfacial defects and promoting crack propagation, ultimately reducing the interfacial adhesion performance between the coating and the cementitious substrate [20].

### 3.5. Coating Adhesion Strength

**Figure 12:**
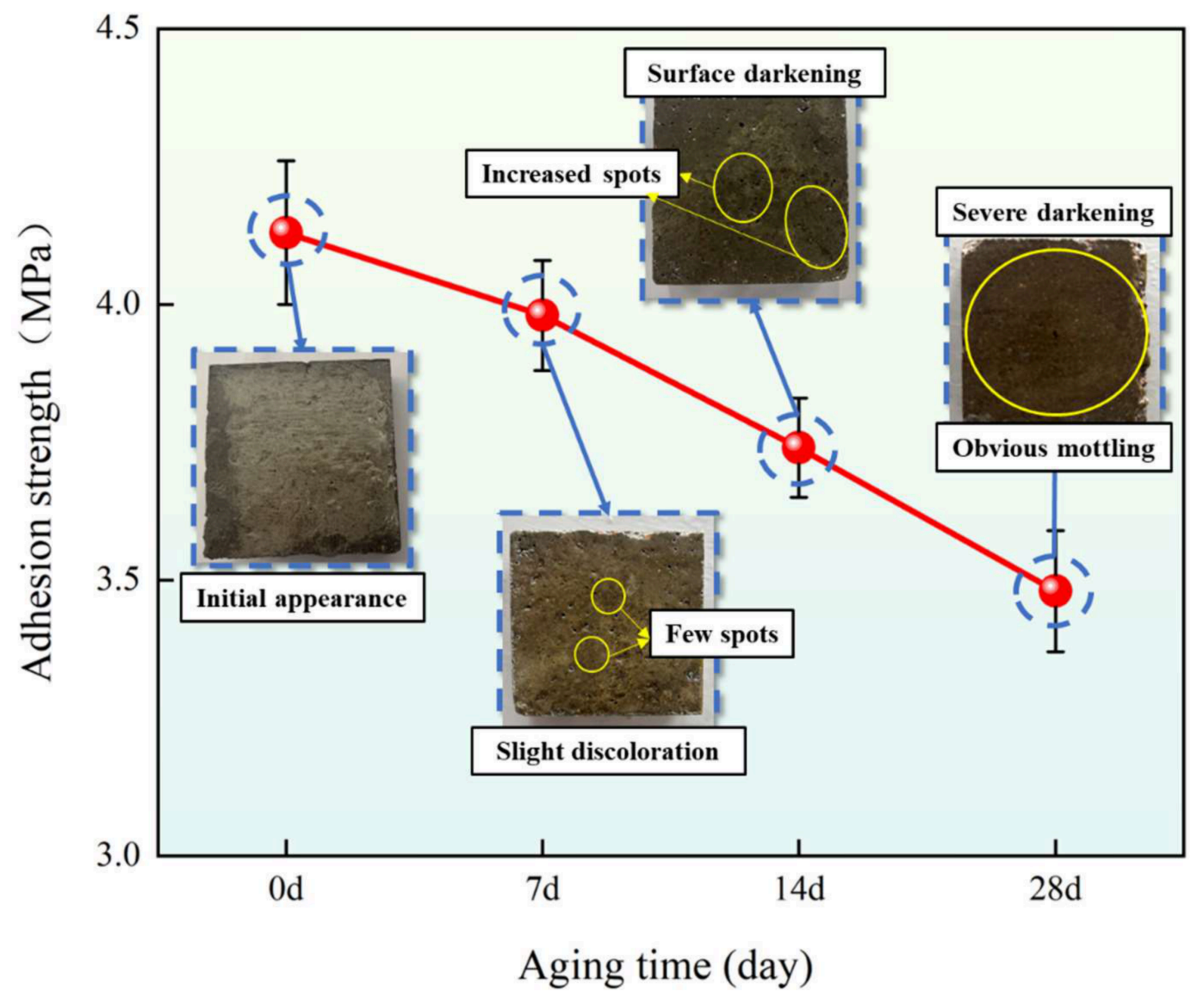
Adhesion strength between epoxy coatings and cementitious substrates at different UV aging durations.

The adhesion strengths of the coatings at different UV aging durations are shown in Figure 12. As shown in the figure and as further indicated by the detailed test results in Table 7, the adhesion strength between the epoxy coating and the cementitious substrate continuously decreased with increasing UV aging duration, from 4.13 ± 0.13 Mpa in the unaged state to 3.48 ± 0.11 Mpa after 28 d of aging, representing a reduction of 15.7% and indicating progressive deterioration of the coating adhesion performance. This trend was consistent with the evolution of the coating nano-adhesion and toughness. With increasing aging duration, the mean nano-adhesion of the coating decreased, the high-adhesion regions gradually diminished, the low-adhesion regions continuously increased, and the spatial variability of coating adhesion increased. Under pull-off loading, these low-adhesion regions were more likely to become initiation sites for debonding and crack propagation [60,61]. Meanwhile, UV aging altered the characteristic molecular structure of the epoxy coating and reduced its toughness and deformation compatibility, making stress concentration and debonding propagation more likely to occur at interfacial defects [20], thereby further reducing the adhesion strength.

**Figure 13:**
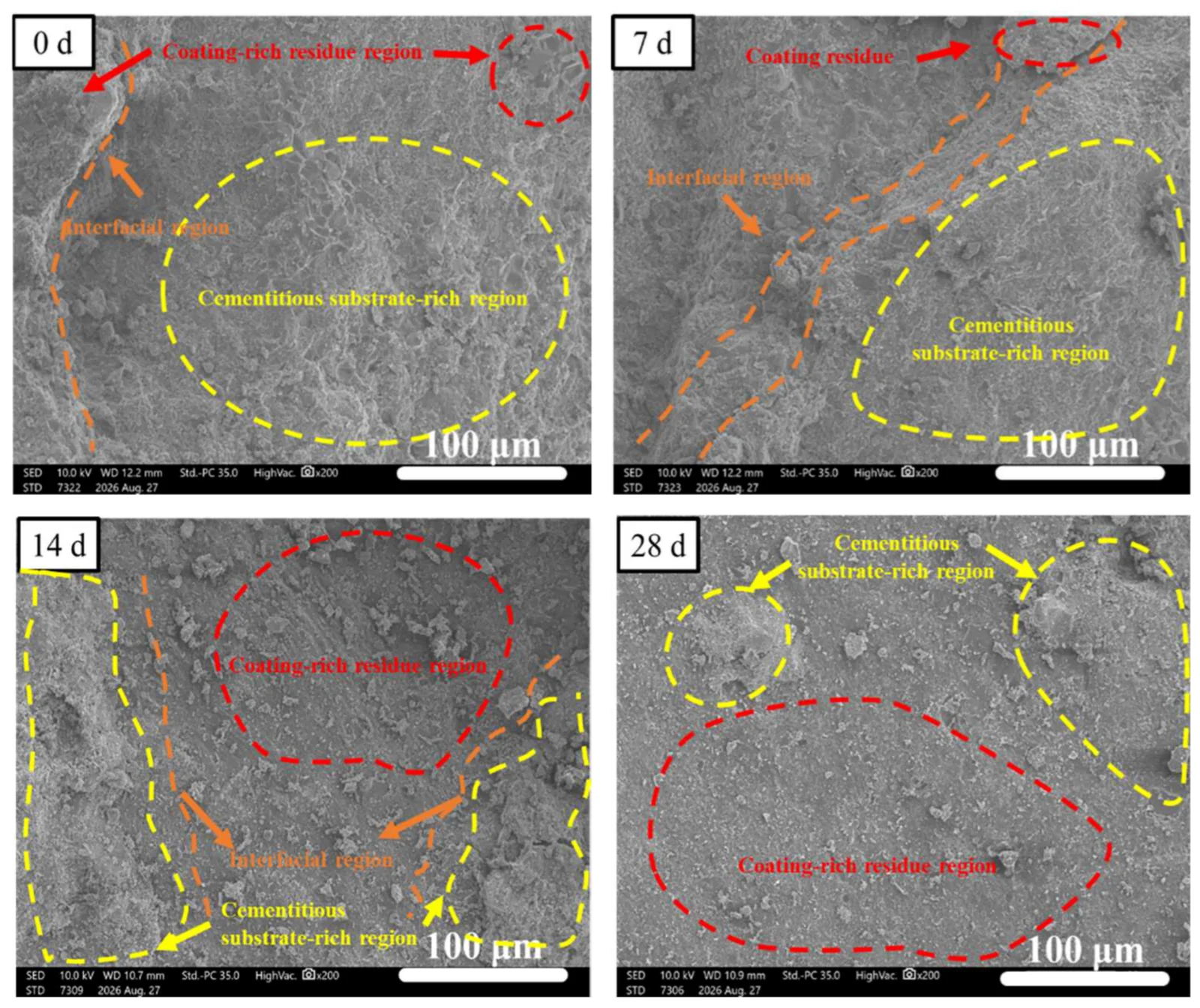
SEM morphologies of the epoxy coating–cementitious substrate interface at different UV aging durations.

**Figure 14:**
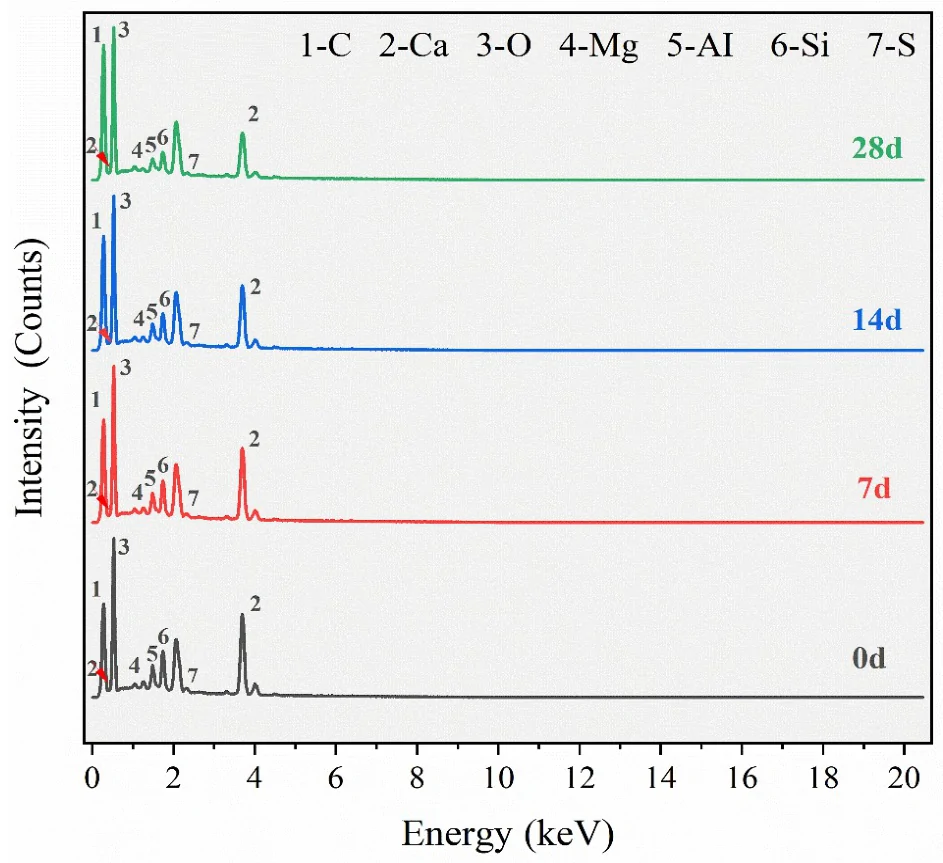
EDS spectra of pull-off fracture surfaces at different UV aging durations.

To further analyze the failure modes of the epoxy coating–cementitious substrate interface at different UV aging durations, typical failure regions after the pull-off tests were examined by SEM and EDS. SEM observations were used to identify the microstructural characteristics of the fracture surfaces, while EDS analysis was performed to evaluate the C signal intensity associated with the epoxy coating and the Ca and Si signal intensities associated with the cementitious substrate [64]. The test results are shown in Figure 13 and Figure 14, respectively. Table 8 and Table 9 present the quantitative EDS results for the specimens at different aging durations, including the weight percentage (wt%) and atomic percentage (Atomic%) of each element. The EDS quantitative results are expressed as the measured value ± the instrument-reported error. Combined analysis of the SEM and EDS results shows that, with increasing UV aging duration, the microscopic morphology and local elemental composition of the fracture surfaces exhibited relatively consistent evolution trends. C, O, Mg, Al, Si, S, and Ca were detected in all four groups of specimens, although their relative contents varied with aging duration. In the SEM image of the unaged specimen, relatively distinct cementitious-substrate-rich regions and interfacial regions were observed, together with only a small amount of residual coating. Correspondingly, the EDS spectrum exhibited relatively strong signals of Ca and Si associated with the cementitious substrate, whereas the C signal associated with the epoxy resin was comparatively weak. Quantitative EDS analysis showed that the wt% values of C, Ca, and Si were 15.35% ± 0.06%, 41.48% ± 0.21%, and 4.32% ± 0.06%, respectively, while their Atomic% values were 26.61% ± 0.10%, 21.55% ± 0.10%, and 3.20% ± 0.04%, respectively. Based on the Atomic% values obtained from the EDS results, the atomic ratios were calculated [65], yielding C/Ca and C/Si ratios of 1.23 and 8.32, respectively. Combined with the SEM observations, these results indicate that failure at this stage occurred mainly at the coating–cementitious substrate interface and within the cementitious substrate. After 7 d of aging, the coating-rich regions in the SEM images increased slightly. Compared with the unaged state, the C signal began to increase, whereas the Ca and Si signals decreased. The wt% and Atomic% of C increased by 32.3% and 23.0%, respectively, while those of Ca decreased by 16.6% and 22.6%, respectively, and those of Si decreased by 3.5% and 10.3%, respectively. Accordingly, the C/Ca and C/Si atomic ratios increased to 1.96 and 11.40, respectively, reflecting an increased contribution of cohesive failure within the coating in the interfacial failure region. After 14 d of aging, the coating-rich characteristics of the fracture surface became more pronounced, accompanied by a further decrease in the Ca and Si signals and a further increase in the C signal. Compared with the initial state, the wt% and Atomic% of C increased by 58.2% and 39.6%, respectively, whereas those of Ca decreased by 28.2% and 36.7%, respectively, and those of Si decreased by 10.0% and 20.6%, respectively. The C/Ca and C/Si atomic ratios further increased to 2.72 and 14.63, respectively, indicating a gradual transition of the failure characteristics toward cohesive failure within the coating. After 28 d of aging, the fracture surface was mainly composed of extensive coating-rich regions together with a small proportion of coating–cementitious substrate interfacial regions. Compared with the unaged state, the C signal increased markedly, whereas the Ca and Si signals decreased substantially. The wt% and Atomic% of C increased by 114.5% and 69.9%, respectively, while those of Ca decreased by 54.8% and 64.2%, respectively, and those of Si decreased by 25.9% and 41.3%, respectively. The C/Ca and C/Si atomic ratios reached 5.86 and 24.05, respectively. Overall, the quantitative EDS results showed a certain correspondence with the morphological evolution of the different enriched regions observed by SEM. With increasing aging duration, the relative proportion of coating-related elements in the analyzed fracture-surface regions increased, whereas that of cementitious-substrate-related elements gradually decreased.

Based on the above analysis, as UV aging progressed, the proportion of exposed cementitious substrate on the pull-off fracture surface gradually decreased, while the area covered by residual coating continuously increased. Consistently, the C signal associated with the epoxy coating in the EDS spectra progressively increased, whereas the Ca and Si signals associated with the cementitious substrate continuously decreased. The weight percentage (wt%) and atomic percentage (Atomic%) of C continuously increased, whereas those of Ca and Si generally decreased. The C/Ca and C/Si atomic ratios increased from 1.23 and 8.32 in the unaged state to 5.86 and 24.05 after 28 d of aging, respectively. These results further indicate that, with increasing coating degradation, the failure path gradually shifted from fracture near the substrate surface toward regions close to the coating–cementitious substrate interface and into the interior of the coating. This observation is consistent with the aforementioned evolution of the mechanical properties and Nano-adhesion. With increasing UV aging duration, the tensile strength and toughness of the coating continuously decreased, indicating a progressive reduction in its load-bearing and energy-dissipation capacities and an increasing tendency toward cohesive failure within the coating. Meanwhile, the decrease in mean Nano-adhesion and the increase in low-adhesion regions indicate further deterioration of the local adhesion performance of the coating, resulting in an expansion of failure-prone regions near the coating–substrate interface.

**Figure 15:**
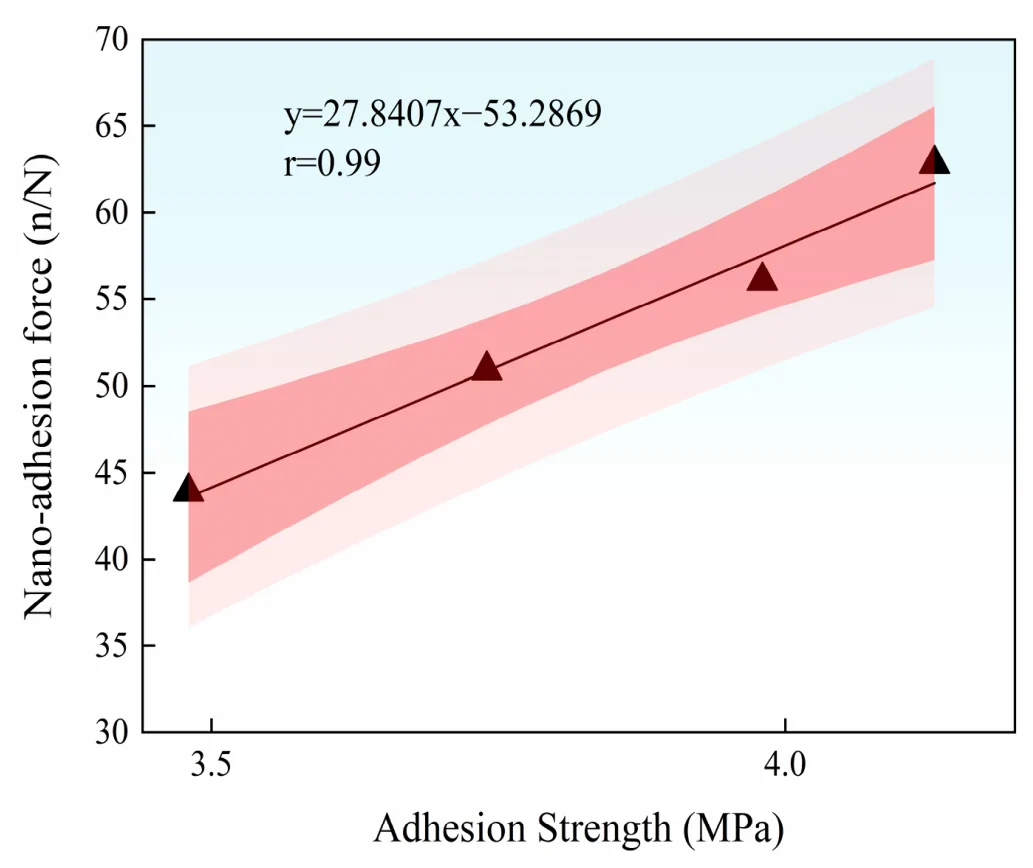
Correlation between mean nano-adhesion and adhesion strength of epoxy coatings.

**Figure 16:**
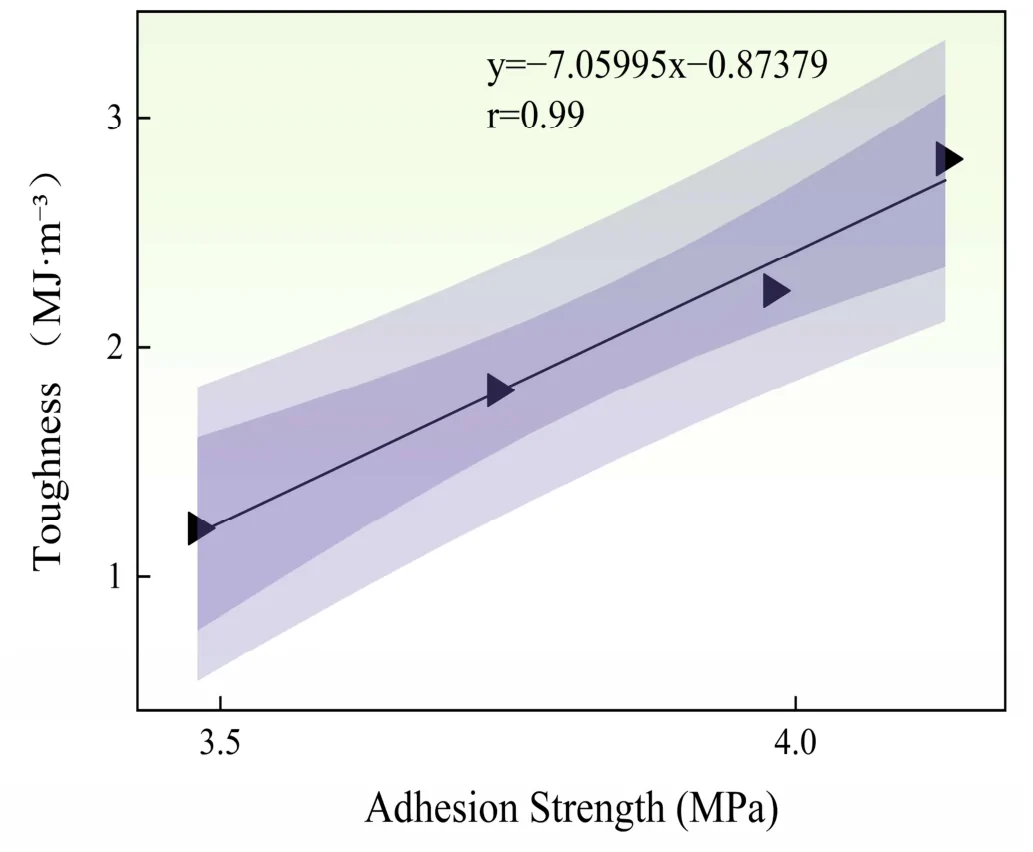
Correlation between toughness and adhesion strength of epoxy coatings.

To further clarify the influence of deterioration in the structure and properties of the coating itself on its adhesion performance, correlation analyses were conducted between the two principal coating performance indicators and the macroscopic adhesion strength, with the results shown in Figure 15 and Figure 16. (The triangles represent the experimental data points, while the dark-red and light-red shaded areas represent the 95% confidence band and 95% prediction band, respectively.) As shown in Figure 15, the mean nano-adhesion of the epoxy coating exhibited a strong positive correlation with the macroscopic adhesion strength, with a Pearson correlation coefficient of 0.99. The mean Nano-adhesion at each aging duration was calculated based on three scanned regions (*n* = 3 scanned regions), indicating a high degree of consistency between their changes during UV aging. The mean nano-adhesion reflects the average level of the local adhesion performance of the coating, whereas the pull-off test characterizes the macroscopic adhesion performance of the coating-cementitious substrate system.

With increasing UV aging severity, the mean Nano-adhesion of the coating gradually decreased, while the high-adhesion regions progressively decreased and the low-adhesion regions increased, indicating a continuous weakening of the local molecular interactions and microscopic adhesion state of the coating, accompanied by greater spatial variability in the adhesion distribution. The increase in these locally weakened regions reduces the continuity of the effective bonded regions within the coating–cementitious substrate system, making local stress concentration more likely during load transfer and promoting the propagation and interconnection of weak regions, ultimately resulting in a decrease in macroscopic adhesion strength [60,61]. Therefore, under UV aging, the decrease in Nano-adhesion is a major factor contributing to the deterioration of macroscopic adhesion strength, and the two exhibit a high degree of consistency in their evolution with UV aging.

As shown in Figure 16, the Pearson correlation coefficient between the coating toughness and adhesion strength was 0.99, indicating a strong positive correlation and a high degree of consistency between their changes during UV aging. Toughness reflects the ability of the coating to absorb and dissipate deformation energy before fracture, as well as its ability to deform compatibly with the substrate when subjected to external loading and interfacial deterioration. With increasing UV aging severity, structural changes occurred in the cured epoxy network, and the coating toughness gradually decreased, thereby weakening its ability to accommodate the deformation of the cementitious substrate and dissipate interfacial stresses [18,19]. When the coating adhesion performance was evaluated under external loading, such as that applied during the pull-off test, stress concentration was more likely to occur at interfacial defects and promote crack propagation. This was detrimental to maintaining the effective load-bearing area and stress-transfer capacity of the interface, thereby exerting an adverse effect on the macroscopic adhesion strength [20,22].

## 4. Conclusions

This study focused on coating deterioration and interfacial failure under intense UV radiation in high-altitude plateau environments. A multiscale approach was employed to investigate the evolution of coating adhesion, tensile strength, and toughness. By combining the characteristic functional groups, Nano-adhesion, and fracture-surface microstructure of the coating, the effects of UV aging on the intrinsic structure and adhesion performance of the organic protective coating were elucidated. The specific conclusions are as follows:

(1) With increasing UV aging duration, the intensities of the bands associated with hydroxyl and carbonyl groups in the epoxy coating increased, while the total surface free energy and its polar component exhibited overall increases. In particular, the polar component increased to 3.9 times its initial value. Meanwhile, the mechanical properties of the coating gradually deteriorated during UV aging, with its toughness decreasing from 2.82 ± 0.052 to 1.21 ± 0.027 MJ·m^−3^. Consequently, the deformation and energy dissipation capacities of the coating under external loading decreased markedly.
(2) The mean nano-adhesion of the epoxy coating continuously decreased with increasing UV aging duration. After 7, 14, and 28 d of aging, the mean nano-adhesion decreased by 10.7%, 18.9%, and 30.1%, respectively, compared with that of the unaged specimen. The nano-adhesion distribution also changed markedly. The high-adhesion regions gradually became more discrete, whereas the low-adhesion regions and locally heterogeneous regions increased. Consequently, the spatial variability of the coating nano-adhesion distribution increased, and the overall adhesion performance deteriorated.
(3) Under the conditions of this study, the macroscopic adhesion strength of the epoxy coating decreased by 15.7% after 28 d of aging. With the progression of UV aging, the failure path gradually shifted from fracture near the substrate surface toward regions close to the coating–cementitious substrate interface and into the coating itself. The mean Nano-adhesion and coating toughness exhibited strong correlations with the adhesion strength. Decreases in these two indicators significantly weakened the coating adhesion performance. Therefore, these indicators and their influencing factors should receive particular attention in the optimized design of organic protective coatings for regions exposed to intense UV radiation.

## Figures and Tables

**Table 1:** Technical properties of the epoxy coating.

Types of Coatings	Solid Content (%)	Surface Drying Time (h)	Viscosity (mPa·s)	Coating Consumption per 3 mm Thickness (kg/m^2^)
Epoxy resin	60	12	5000	0.7~1.0

**Table 2:** Contact angles of epoxy coatings at different aging durations (°).

Aging Time	Deionized Water	Formamide	Diiodomethane
0 d	82	71	52
7 d	74	66	47
14 d	64	57	43
28 d	51	49	34

**Table 3:** Surface tension and components of the three probe liquids [54].

Liquid	γ^LW^ (mN/m)	γ^p^ (mN/m)	γ^Total^ (mN/m)
Water	21.8	51	72.8
Diiodomethane	50.8	0	50.8
Formamide	39	19	58

**Table 4:** Surface free energies and components of coatings.

Aging Time	γ^LW^ (mJ/m^2^)	γ^p^ (mJ/m^2^)	γ^Total^ (mJ/m^2^)
0 d	29.01	4.33	33.34
7 d	30.66	6.92	37.58
14 d	32.50	11.20	43.70
28 d	35.07	16.86	51.93

**Table 5:** Average Nano-adhesion of epoxy coatings at different aging durations.

Aging Time	0 d	7 d	14 d	28 d
Average Nano-adhesion force (nN)	62.8 ± 1.80	56.05 ± 2.60	50.9 ± 2.35	43.9 ± 2.20

**Table 6:** Tensile properties and toughness of epoxy coatings at different aging durations.

Aging Time	Maximum Stress (Mpa)	Maximum Strain	Toughness (MJ·m^−3^)
0 d	4.79 ± 0.065	0.83 ± 0.010	2.82 ± 0.052
7 d	4.52 ± 0.061	0.718 ± 0.011	2.25 ± 0.047
14 d	3.96 ± 0.054	0.628 ± 0.007	1.81 ± 0.033
28 d	3.29 ± 0.045	0.445 ± 0.008	1.21 ± 0.027

**Table 7:** Pull-off adhesion strength of epoxy coating–cementitious substrate systems at different aging durations.

Aging Time	0 d	7 d	14 d	28 d
Adhesion- strength (Mpa)	4.13 ± 0.13	3.98 ± 0.10	3.74 ± 0.09	3.48 ± 0.11

**Table 8:** Quantitative EDS elemental analysis of specimens aged for 0 d and 7 d.

Element	0 d		7 d	
	wt (%)	Atomic (%)	wt (%)	Atomic (%)
C	15.35 ± 0.06	26.61 ± 0.10	20.31 ± 0.04	32.72 ± 0.07
O	35.36 ± 0.17	46.01 ± 0.20	37.45 ± 0.14	45.28 ± 0.15
Mg	0.56 ± 0.03	0.48 ± 0.02	0.57 ± 0.02	0.45 ± 0.02
Al	2.02 ± 0.05	1.56 ± 0.03	1.99 ± 0.03	1.43 ± 0.02
Si	4.32 ± 0.06	3.20 ± 0.04	4.17 ± 0.05	2.87 ± 0.03
S	0.91 ± 0.04	0.59 ± 0.02	0.93 ± 0.03	0.56 ± 0.02
Ca	41.48 ± 0.21	21.55 ± 0.10	34.58 ± 0.17	16.69 ± 0.08

**Table 9:** Quantitative EDS elemental analysis of specimens aged for 14 d and 28 d.

Element	14 d		28 d	
	wt (%)	Atomic (%)	wt (%)	Atomic (%)
C	24.29 ± 0.05	37.15 ± 0.08	32.92 ± 0.07	45.21 ± 0.11
O	38.72 ± 0.15	44.46 ± 0.17	42.08 ± 0.19	43.38 ± 0.18
Mg	0.54 ± 0.02	0.41 ± 0.02	0.52 ± 0.03	0.35 ± 0.02
Al	1.90 ± 0.04	1.29 ± 0.02	1.67 ± 0.03	1.02 ± 0.02
Si	3.89 ± 0.05	2.54 ± 0.03	3.20 ± 0.04	1.88 ± 0.03
S	0.89 ± 0.03	0.51 ± 0.02	0.87 ± 0.04	0.45 ± 0.02
Ca	29.77 ± 0.18	13.64 ± 0.08	18.74 ± 0.14	7.71 ± 0.06

## Data Availability

The data supporting the reported results in this study are available within the article. Further inquiries can be directed to the corresponding author.
